# Supplementary material for: The Use of the Bolk Model for Positive Health and Living Environment in the Development of an Integrated Health Promotion Approach: A Case Study in a Socioeconomically Deprived Neighborhood in The Netherlands
Source: Int J Environ Res Public Health. 2022 Feb 21;19(4):2478. doi: 10.3390/ijerph19042478 (PMC8879013; doi:10.3390/ijerph19042478)
Supplement: Supplementary file 1 [file ijerph-19-02478-s001.zip › ijerph-1563523-supplementary/S1_Interview guide phase 1 mapping needs and strengths.pdf]

Supplementary Material, S1: Interview guide phase 1 mapping needs and strengths

Questions with a dash in front of the question are optional follow-up questions

General

Introduction of the researcher, explanation and aim of the research, duration 45-70 minutes, privacy statements.

Personal information

Information regarding personal data of the respondent:

- May I ask you how old you are?
- How long have you lived in the Venserpolder district?
- What kind of work do you do in daily life?

Bodily functions and mental well-being

What is health to you?

Do you feel healthy? What do you need to feel healthy?

Do you feel like you are in control of your health?

How much exercise do you have?

- How do you move around?

- Do you walk a lot?

How does living in Venserpolder affect your health?

How do you feel about the health of your neighbors?

- Do you feel that people in this neighborhood feel healthy?

- Do you feel that people are actively involved with their health?

Daily functioning

What keeps you busy during the day? Is that going well for you?

- What should be done to make that easier?

- Who or what could help you with that?

Meaning

What gives meaning to your life?

- Do you have a goal for the future?

- Do you have a specific task? - A reason?

Who or what helps with this? - Who or what makes it difficult?

- Do you think you can be helped with that?

How would you describe your culture?

- How does this affect your daily life and health?

- Do you feel at home in Venserpolder?

Quality of life

What do you think about the quality of life?

- What is important in your life?

- Does the neighborhood has influence on this?

Social participation

How do you feel about the contact you have with other people? - and with neighbors?

If you are sick, who will take care of you? - also neighbors?

Social living environment

How do neighbors interact with each other?

- Do neighbors come to visit each other?

- Where do people meet in Venserpolder?

- Is there a sense of community in Venserpolder?

- How does this affect your health?

Do you feel that people are lonely or stressed in the neighborhood?

Do you feel safe in the neighborhood?

Would you like to improve something in a social sense in Venserpolder?

Physical living environment

What do you think of the houses and buildings in Venserpolder?

- Do you think the neighborhood looks nice?

What do you think of the greenery in the neighborhood?

Do you think Venserpolder is a clean neighborhood?

What do you think of the traffic in Venserpolder?

Are you experiencing nuisance in the neighborhood?

- How does this affect your health?

Would you like to improve something in terms of the physical environment?

Services and facilities

What do you think of the health facilities in Venserpolder?

What do you think of the community centers?

- What do you think of the community activities in Venserpolder? Do you miss anything?

- Do you participate in community activities? Why / why not?

- If you could organize activities yourself, how would you do that?

What are the possibilities for exercising or sports in Venserpolder?

For what reasons do you generally go out of the neighbourhood?

Can you always eat healthy?

- Where do you buy healthy food?

Would you like to improve something in terms of services and facilities in Venserpolder?

Sense of community

What do you like most about living in Venserpolder?

- Can you name three things?

What do you really not want to miss from your neighborhood?

Ideas

Do you have any suggestions on how we can reach people in the neighborhood?

Closing

Thank you. Ask what the respondent thought of the interview. Whether he or she has any suggestions.
