# Supplementary material for: The Use of the Bolk Model for Positive Health and Living Environment in the Development of an Integrated Health Promotion Approach: A Case Study in a Socioeconomically Deprived Neighborhood in The Netherlands
Source: Int J Environ Res Public Health. 2022 Feb 21;19(4):2478. doi: 10.3390/ijerph19042478 (PMC8879013; doi:10.3390/ijerph19042478)
Supplement: Supplementary file 1 [file ijerph-19-02478-s001.zip › ijerph-1563523-supplementary/S2_Interview guide process evaluation.pdf]

Supplementary Material, S2: Interview guide process evaluation

Interview guide Dutch professionals

Name respondent: Date:
Organisation respondent: Location:
Contact information respondent:
Resident: Yes/No

Attendees
Name interviewer:
Name transcriber:

Materials
- ❑ Voice recorder
- ❑ Interview guide in the informal T-form and the polite V-form
- ❑ Pen & paper
- ❑ Chocolate

Goal of the interview:
To gain insight in to what extent a mind shift has occurred within the respondent and which factors attributed to- or hindered this change process.

Introduction
Thank you for having me and that you made time to do an interview with you. I will explain again what I do and what the aim is of this interview. Currently I am doing research for the Louis Bolk Institute to gain knowledge on how the health approach works in the project Venserpolder Groeit & Leeft and how we can improve it.

All collected data will be processed confidentially and will only be at our disposal. I want you to ask to be as honest as possible, so that we can work together to improve the neighbourhood. Often we can learn a lot from experiences, especially negative experiences. The results will be processed anonymously, so you may speak freely in confidence. Also, it helps if you can mention examples. If you do not know an example, that is okay of course, but do not hesitate to elaborate on a specific experience you had. If you have any questions during the interview, you can always ask them and every moment you can decide that you do not want to proceed with the interview. Afterwards, we will send you a summary of the interview, in order for you to check if we interpreted the things you said correctly. Do I have your consent to record this conversation?

Introduction questions:
I: How long have you been living in Venserpolder?
I: How did you get involved in this project?
I: Can you describe your role in the project? What do you do for the project?

[Urgency]
I: Do you think there is a sense of urgency to change the course of this neighbourhood when it comes to tackling health problems?
Why/why not?

Probing:
I: Do you think there is an urgency to implement an integrated health approach?
I: Why does the current health approach not work according to you?
I: Why are you willing to invest energy in this project?

[Understanding]
I: Can you explain what Positive Health is according to you?

[Acceptance]
[Personal]
I: How do you feel about the concept Positive Health?
I: What are the disadvantages of the concept Positive Health according to you?
I: What do you think about the applicability of the concept Positive Health in Venserpolder? Why/why not?
I: Do you think it can cause the change which is required?

[Integration]
I: Did your view on health change after getting to know the concept of Positive Health?
I: How did Positive Health help you during this project?
Probing:
How does it influence your decisions making?
How does it influence your approach in this project?

[Team]
I: For whom do you think it is important to know what Positive Health is?
I: Do you also recognize a change in others?
I: Do you think that everyone has enough understanding of the definition of Positive Health?
I: What is your opinion on how the communication takes place about Positive Health?

[Personal barriers]
I: Which obstacles do you experience when working from a Positive Health perspective?
I: Which rules/policy are limiting you in your actions according Positive Health?
I: What did you learn from this project?
I: What would you like to learn more?
I: What gives you the energy to carry on?
I: What are the things that demotivate you in this project?

[Composition Guiding Coalition]
I: Which competences does the project team have? What are the most important competences in the project team?
I: Do you have the idea that all parties involved are taking you seriously?
I: Which qualities or expertise do you miss in the project team?
I: Are there organisations who were not involved in the project, but of which you see their added value?
Can you tell me why that is?
I: Are there people who stopped during the project?
And what was/is the effect of this?

[Collaboration project team]
I: How do you think Positive Health/broader view on health influences the collaboration in the project?
How do you recognise this in practice?
I: How would the collaboration in the project look like without the Positive Health perspective/integrated vision?

I: Can you tell me something about the collaboration within the project team?
Are you content with the collaboration?
Do you think the collaboration is effective?
What problems do you run into?

What are the things that contributed to the collaboration?
How could this be improved?

[Collaboration pilots]
I: What is your opinion on how connections are established between pilots?
(Pilots: to what extent do you feel part of a whole?)

[Outcomes project]
I: When is the project successful according to you?
I: How can we achieve that?
I: What do you think about the progression of the integration of the residents' needs?
I: What is your opinion on the quality of the pilots?
What contributes to the quality of the pilots?

I: Do the pilots reach their goal according to you?
I: Are there people who you did not reach of who you had hoped to reach?
Why is that?

[Sustainability]
I: How do you think we can ensure what we keep solving new problems in the right way? From a Positive Health perspective?
I: Do you think that there is a permanent change in people's view on the concept of health?
I: Do you think that the change which is initiated is sustainable?
I: What do you think is needed to establish sustainable change?

I: What recommendations would you like to leave for future projects?

Interview guide Dutch residents

Name respondent: Date:
Organisation respondent: Location:
Contact information respondent:
Resident: Yes/No

Attendees
Name interviewer:
Name transcriber:

Materials
- ❑ Voice recorder
- ❑ Interview guide in the informal T-form and the polite V-form
- ❑ Pen & paper
- ❑ Chocolate

Goal of the interview:
To gain insight in to what extent a mind shift has occurred within the respondent and which factors attributed to- or hindered this change process.

All collected data will be processed <u>confidentially</u> and will only be at our disposal. Several questions will be <u>obvious questions</u>, but for me it is very important to also write down these obvious things. I want you to ask to be as <u>honest</u> as possible, so that we can work together to improve the neighbourhood. Often we can learn a lot from experiences, especially <u>negative</u> experiences. The

results will be processed anonymously, so you may speak freely in confidence. Also, it helps if you can mention examples. If you do not know an example, that is okay of course, but do not hesitate to elaborate on a specific experience you had. If you have any questions during the interview, you can always ask them and every moment you can decide that you do not want to proceed with the interview. Afterwards, we will send you a summary of the interview, in order for you to check if we interpreted the things you said correctly. Do I have your consent to record this conversation?

Introduction questions:

I: How long have you been living in Venserpolder?
I: How did you get involved in this project?
I: What is the aim of Venserpolder Groeit & Leeft according to you?
I: Can you tell me what you do in the project?
I: How does that relate to the bigger picture?

[Urgency]
Aim of the following questions: Is there a sense of urgency to invest in change?

I: What are your experiences with care and well-being in Venserpolder?
I: What changes would you like to see in the neighbourhood?
I: What is the reason that you want to be involved in this project?

[Understanding]
Aim of the following question: What is the interpretation of the concept Positive Health of the respondent?

I: Can you explain what Positive Health is according to you?

[Acceptance]
Aim of the following questions: To what extent does the respondent agree with the concept of Positive Health and accepts this as her/his own view?

I: What do you think are the positive aspects of Positive Health?
I: What do you think are the negative aspects of Positive Health?
I: Do you think that Positive Health can initiate change in Venserpolder?
  Why/why not?
I: How do you think could Positive Health be helping Venserpolder?

[Integration]
Aim of the following questions: To what extent and how is behaviour influenced by the concept of Positive Health?
I: Has your view on health changed after getting to know the concept of Positive Health?
I: Did it have influence on how you think and act?
I: How does Positive Health help you?

  Probing:
  How does it influence your decisions making?
  How does it influence your approach in this project?

[Team]
I: Do you also recognize a change in others with respect to thoughts and actions?

[Communication of the vision/aim of the project and Positive Health]
Aim: Is there a clear shared vision for the project? How did this shared vision came into being?

I: Can you explain me what the goal is of the project Venserpolder Groeit & Leeft according to you?
I: How is Positive Health explained to you?
I: Did you understand it at once?

[Personal barriers]
Aim of the following questions: What are the barriers to make this project successful and to realise an integrated health approach?

I: What would you like to achieve in Venserpolder?
I: Do you need help with that or do you know how to achieve that?
I: What did you learn from this project?
I: What would you like to learn more?
I: What gives you the energy to carry on?
I: What are the things that demotivate you in this project?
Or I: What are the things that costs you energy?

[Project team]
Aim of the following questions: What are the strengths and points for improvement in the project team?

[Composition]
I: What do you think are the strengths of the project team?
I: What do you think are the weaknesses of the project team?
I: There have been many changes in the project team, like interns. What is your opinion about that?
Or I: Can you describe what kind of effect it had on the project?

[Bottom-up approach]
Aim of the following questions: How does the bottom-up approach takes place?
I: Do you know who the ambassador is of the Green and Health project?
I: What is her contribution to the Green and Health project?
    What else could her contribution be?
I: Do you have the idea that you are taken seriously?
    Why is that?
I: Who is the owner of the Coffee Hour according to you? Or I: Who decides how het Coffee Hour looks like?
I: Is the Green and Health project now exactly how you have had imagined it?
    Why/why not?
I: What do think about how the bottom-up approach is implemented? Do you think the residents' opinions are being heard?
    Why/why not?
I: How could that be improved?

[Collaboration]
Aim of the following questions: How does the integrated approach influences the collaboration? Is the collaboration successful?

I: How do you think Positive Health/broader view on health influences the collaboration in the project?
    How do you recognise this in practice?
I: How would the collaboration in the project look like without the Positive Health perspective/integrated vision?
I: To what extent do you collaborate with de Groei en Bloei tuin?

[Connection between pilots]
I: How does the Green and Health pilot fit into the bigger picture?

I: Do you feel part of the whole?
I: Are you content with the collaboration?
    Why/why not?
    What problems do you run into?
    What are the things that contributed to the collaboration?

I: Are there organisations who were not involved in the Green and Health pilot, but of who you would have liked to be involved?
    Can you tell me why that is?

[Outcomes project]
Aim: Do the outcomes of the project correspond with the goal?
I: When is Venserplder Groeit & Leeft successful according to you?

I: What would you like to achieve with Venserplder Groeit & Leeft?
I: What needs to be done in order to achieve that?

[Scope]
Aim: What group of people do we need to reach and is it successful?

I: Are you content with the scope of the Green and Health project?
I: How can we reach more people according to you?
I: Do you think everybody in Venserpolder knows what Venserpolder Groeit & Leeft is?
I: What group of people would you like to reach with this health project?
Or I: For who do you think we can mean the most with this health project?
I: How could we ensure that we reach them according to you?

[Sustainability]
Aim: To what extent a sustainable integrated approach created in the neighbourhood?

I: Do you think that there is a permanent change in people's view on the concept of health?
I: If you want to organise something in the neighbourhood again in the future, do you now know how to approach it?
I: Do you think that the change which is initiated is now sustainable?
I: What do you think is needed to establish sustainable change?

I: What recommendations would you like to leave for future projects?
