# Supplementary material for: The Use of the Bolk Model for Positive Health and Living Environment in the Development of an Integrated Health Promotion Approach: A Case Study in a Socioeconomically Deprived Neighborhood in The Netherlands
Source: Int J Environ Res Public Health. 2022 Feb 21;19(4):2478. doi: 10.3390/ijerph19042478 (PMC8879013; doi:10.3390/ijerph19042478)
Supplement: Supplementary file 1 [file ijerph-19-02478-s001.zip › ijerph-1563523-supplementary/S3_Description of the HPPs.pdf]

Supplementary Material, S3: Description of the HPPs

Below is a short description of the HPPs as summarized in Table 2 of the manuscript.

1. Coffee hour in the Health care centre

This HPP is described in the main text in detail.

2. Digital connecting

This HPP was the result of a desire of residents to better know which activities are being organised in the neighbourhood. Care organisations expressed a desire to be better informed about informal care options in the neighbourhood. In this HPP data on activities and organisations was collected and used as input for a digital portal, accessible to all residents in the local public library. This user friendly portal helps people find activities and connect with care organisations. The number of people using this particular portal in the local library was not assessed. The 21 portals throughout Amsterdam are visited over 60.000 times each month (April 2019).

3. Healthy shopping area

Residents expressed the desire for the availability of more healthy products in the local shopping area in Venserpolder. This idea was not turned into an actual HPP because there was no organisation supporting this idea. The project team hired a project developer to make a plan for a healthy shopping area. This plan was supported by the local government and is currently become part of the process for reorganisation of the shopping area.

4. Green and Health workshops

A group of women have established a vegetable community garden in the neighbourhood some years ago. This community garden plays an important social function in the neighbourhood. In this HPP six workshops were organised by some of the women in this community garden for other women with several themes: teaching them how to eat healthy, how to make small gardens around the house, how to conserve vegetables, how to read food labels, how to make a garden on a balcony and about flowers. On average 8 women participated in each of the 6 workshops.

5. Fire and Eat adventure workshops

Residents felt that there is a need for kids to understand more about healthy eating. In this HPP 16 workshops 1,5 hour each were organised by a local nature expert for kids (8-12 years old) in the community garden. Themes were: fire making skills, cooking vegetables, eating together and learning to eat new vegetables. The workshops were attended by 12 kids on average, with a total of 26 different kids.

6. Man power

There was interest to organise activities for men in the neighbourhood. A community centre was willing to contribute to this HPP but no (male) residents were found that were willing to take the lead. This HPP was cancelled.

7. Accessibility

Several residents expressed problems with access to roads, and building for people in wheelchairs. In this HPP two neighbourhood tours with professional organisations were used to identify issues of accessibility. These issues were presented to the local government. During this HPP several walkways were adapted. A connection with a local neighbourhood

committee with regular meetings with the local government was established. One person with special needs was invited in this committee to represent this theme.

8. Thematic health events

Residents were interested to share knowledge about health and personal development. A group of residents was formed which organized events with health related themes such as: positive health, exercise, and cultural aspects of health. Each event was attended by 20-50 people. The project team supported the HPP in writing funding requests for the events.
